# POLIII-derived non-coding RNAs acting as scaffolds and decoys

**DOI:** 10.1093/jmcb/mjz049

**Published:** 2019-06-01

**Authors:** Hendrik Täuber, Stefan Hüttelmaier, Marcel Köhn

**Affiliations:** 1 Institute of Molecular Medicine, Section for Molecular Cell Biology, Faculty of Medicine, Martin Luther University Halle-Wittenberg, Charles Tanford Protein Centre, Kurt-Mothes-Str. 3a, 06120 Halle, Germany; 2 Julius Bernstein Institute of Physiology, Faculty of Medicine, Martin Luther University Halle-Wittenberg, Charles Tanford Protein Centre, Kurt-Mothes-Str. 3a, 06120 Halle, Germany

**Keywords:** ncRNA, POLIII, RBP scaffold, RBP decoy

## Abstract

A large variety of eukaryotic small structured POLIII-derived non-coding RNAs (ncRNAs) have been described in the past. However, for only few, e.g. 7SL and H1/MRP families, cellular functions are well understood. For the vast majority of these transcripts, cellular functions remain unknown. Recent findings on the role of Y RNAs and other POLIII-derived ncRNAs suggest an evolutionarily conserved function of these ncRNAs in the assembly and function of ribonucleoprotein complexes (RNPs). These RNPs provide cellular `machineries’, which are essential for guiding the fate and function of a variety of RNAs. In this review, we summarize current knowledge on the role of POLIII-derived ncRNAs in the assembly and function of RNPs. We propose that these ncRNAs serve as scaffolding factors that `chaperone’ RNA-binding proteins (RBPs) to form functional RNPs. In addition or associated with this role, some small ncRNAs act as molecular decoys impairing the RBP-guided control of RNA fate by competing with other RNA substrates. This suggests that POLIII-derived ncRNAs serve essential and conserved roles in the assembly of larger RNPs and thus the control of gene expression by indirectly guiding the fate of mRNAs and lncRNAs.

## Features of POLIII-derived small ncRNAs

Transcription by RNA Polymerase III (POLIII) is a unique type of RNA synthesis. POLIII-synthesized transcripts are typically rather small or medium sized (up to ~300 nts) and exclusively constitute non-protein coding RNAs (ncRNAs). Three modes of POLIII transcription have been described based on the genomic location and motifs facilitating transcription. Types I and II POLIII-transcription is usually termed ‘internally initiated’ since major regulatory transcription motifs are located within the transcribed sequence itself. The majority of internally initiated transcripts include the 5S rRNA (type I) and tRNA genes (type II). 5S rRNA transcription is initiated by binding of TFIIIA to the internal control region consisting of distinct short sequence motifs termed A-, IE-, and C-block. However, type II POLIII synthesis is initiated by TFIIIC associating with the short intragenic A- and B-boxes. Type III POLIII genes are usually referred to as ‘externally initiated’. The transcription of these relies on motifs (e.g. TATA-box) outside of the transcribed sequence for efficient synthesis (e.g. U6 snRNA). The modes and mechanisms of POLIII-mediated transcription have been studied extensively and were reviewed previously (Canella et al., 2010; White, 2011; Arimbasseri and Maraia, 2016).

Irrespective of their mode of transcription, POLIII-derived ncRNAs share features, which influence their structure and association with RNA-binding proteins (RBPs). POLIII-derived ncRNAs usually start with purine nucleotides and guanosine at the +1 position is greatly favored over adenosine (e.g. in Y5 or H1 RNAs; Ma et al., 2014). This terminal nucleotide can be modified by the methylphosphate capping enzyme MEPCE (e.g. shown for 7SK and U6) resulting in severely increased RNA stability (Shumyatsky et al., 1993; Jeronimo et al., 2007). Furthermore, a higher content in purine bases at the 5′-end enables the formation of more stable internal RNA duplexes and increased resistance toward exonucleases. All nascent POLIII-transcripts end with an oligo-uridine terminator sequence. Four consecutive uridine nucleotides are sufficient for the proper 3′-end termination of POLIII-generated transcripts (Nielsen et al., 2013; Gao et al., 2018). The terminator sequence is the primary binding site for the La-protein (Gottlieb and Steitz, 1989). The major role of La is to protect the nascent transcript from degradation and to increase the transcriptional output of the gene by enabling multiple rounds of transcription from one gene (Maraia et al., 1994; Dieci et al., 2013). Notably, some POLIII-transcripts are processed at their 3′-end following their synthesis. This trimming removes the oligo-uridine sequence and consequently impairs the association of the La protein (e.g. Y RNAs and 5S; Simons et al., 1996; Ciganda and Williams, 2011). The majority of trimmed POLIII-transcripts are cytoplasmic. Therefore it is likely that the La-binding site has to be removed to facilitate nuclear export of these transcripts. In support of this view, the La protein was shown to inhibit the nuclear export of Y RNAs (Simons et al., 1996). Accordingly, it appears likely that cytoplasmic functions of these ncRNAs are largely La-independent.

## 7SL scaffolds the signal recognition particle

**Figure 1:**
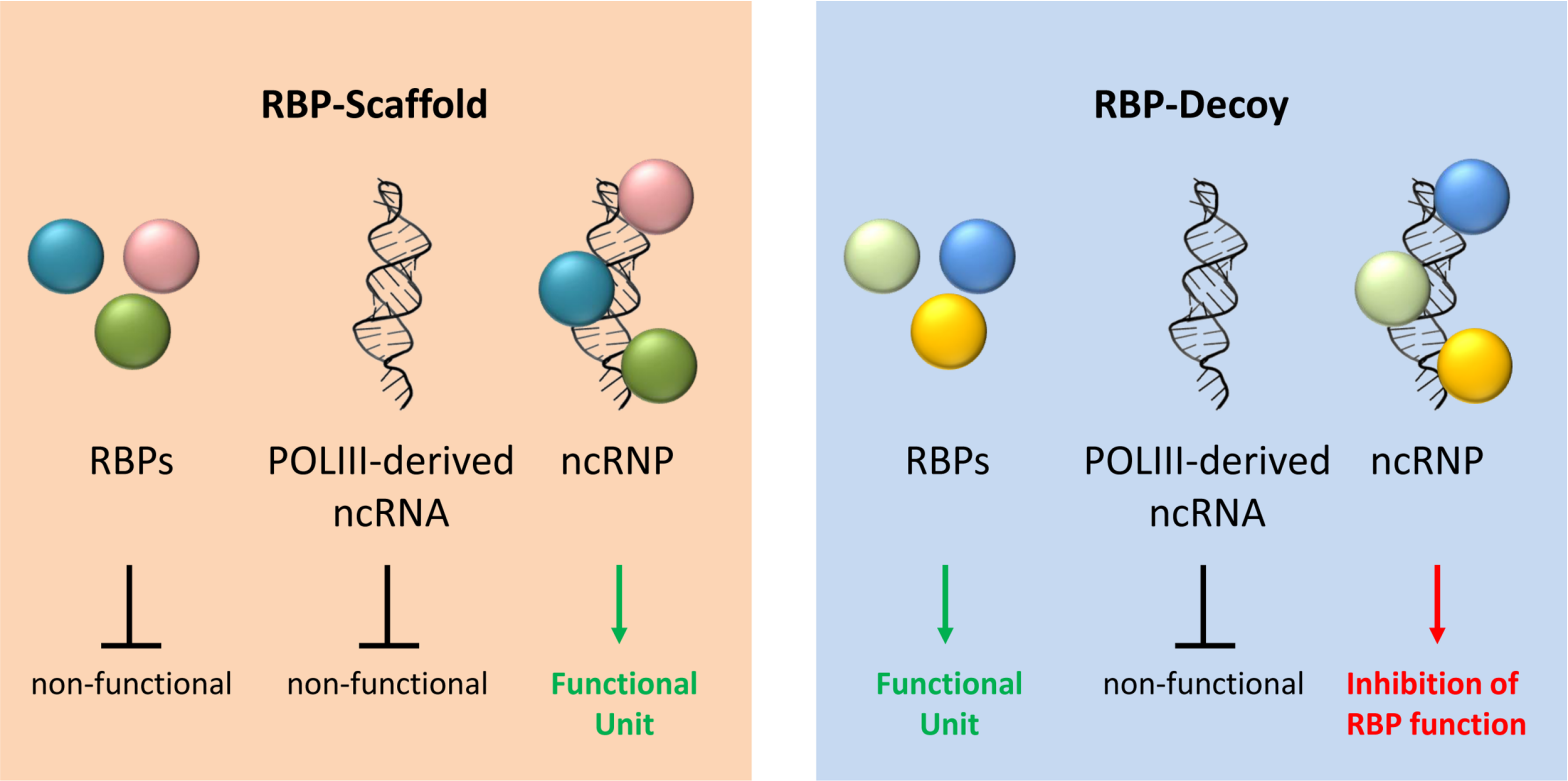
Function of ncRNPs. NcRNA and RBP form a scaffolding complex that is able to perform functions that none of the components can fulfill individually. In contrast, ncRNAs can also act as molecular decoy to inhibit RBP function.

The cellular function of POLIII-generated ncRNAs appears to be tightly linked to their associated RBPs. However, the cellular function of a formed ncRNA containing ribonucleoprotein (ncRNP) is not pre-determined. The best described role of POLIII-derived ncRNAs is to act as molecular scaffold guiding or ‘chaperoning’ the formation of ncRNPs. Importantly, this mode of function implies that neither the associated RBPs nor the ncRNA facilitate a molecular function alone. Thus, the formed ncRNP is the solely functional unit (Figure 1). Several mechanisms have been proposed why RBPs and ncRNAs need to form such comparatively small ncRNPs. One such mechanism is that upon binding both, ncRNAs and RBPs undergo structural transformations. These are likely to induce conformational changes promoting the recruitment of additional co-factors (e.g. SRP19 in the 7SL-RNP, see below). The ncRNA can furthermore trigger the interaction of two RBPs that normally cannot interact on their own (e.g. 7SK RNP). On the cellular level ncRNAs can also direct RBPs to specific subcellular locations and thus facilitate guided localization (e.g. protein targeting to the endoplasmic reticulum (ER) by 7SL). Small ncRNAs, moreover, interact with RNA-dependent enzymes (e.g. helicases and fibrillarin) and promote their association with substrates (e.g. snoRNAs).

With a size of ~300 nts, the human 7SL ncRNA is one of the largest ncRNAs synthesized by POLIII. It folds into a typical structure that is characterized by helical regions forming a smaller (Alu) domain and a larger (S) domain (Pool, 2005). Each part of the ncRNA is able to recruit different sets of proteins that finally assemble the signal recognition particle (SRP). The main function of the SRP is to facilitate protein synthesis at the ER membrane. The SRP recognizes signal peptides emerging from the ribosome during translation, stalls the translation process, and targets the ribosome to the ER membrane, where local translation can resume into the ER lumen. This process ensures that proteins that have to be secreted or are localized to the membrane are translated into the ER and can thereby be distributed properly. The Alu domain of 7SL is the preferential binding site for the SRP14/SRP9 heterodimer. The primary function of the protein bound Alu domain is to inhibit translation elongation after signal recognition (Siegel and Walter, 1988). This is achieved by blocking the elongation factor binding site in the ribosome itself (Halic et al., 2004; Bousset et al., 2014). SRP19 directly associates with 7SL in the S domain and this interaction is a prerequisite for SRP54 to associate with the SRP (Wild et al., 2001). The S domain-associated SRP54 can recognize the signal peptide of the nascent amino acid chain at the exit site of the ribosome (Gowda et al., 1997). Furthermore, SRP54 mediates the interaction of the SRP with the SRP receptor at the ER membrane in a GTP-dependent manner and thereby connects the Ribosome to the translocon channel at the ER (Pool et al., 2002). The SRP68/SRP72 heterodimer is responsible for restructuring the 7SL ncRNA to enable proper interaction of the SRP with the ribosome (Grotwinkel et al., 2014). Furthermore, SRP68/SRP72 is required for the pre-SRP complex to leave the nucleus after transcription (van Nues et al., 2008).

The SRP represents a unique example for the scaffolding role of POLIII-derived ncRNAs in the formation of functional RNPs. Due to the hierarchical recruitment of each 7SL-associated protein it is ensured that 7SL and thereby the SRP adopts a structure that finally results in the recruitment of SRP54 and the formation of fully functional SRPs. Importantly, some RBPs assure structural premises for the proper assembly and function of SRPs (e.g. SRP19, SRP68/SRP72).

## 7SK as decoy factor for P-TEFb

**Figure 2:**
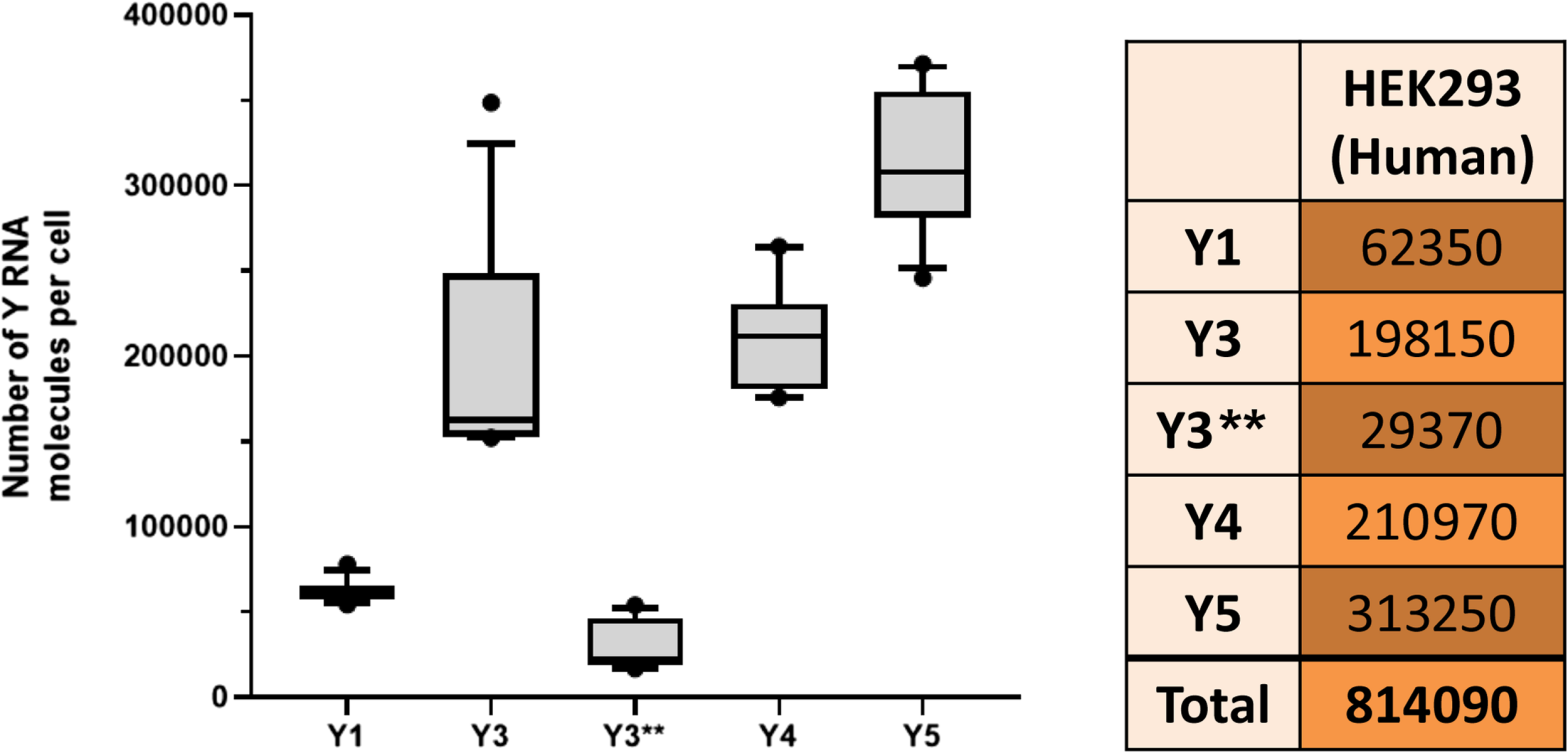
Quantification of Y RNA levels. Human Y RNAs were synthesized by *in vitro* transcription. Cellular total RNA was extracted from HEK293 cells using TRIZOL (Sigma-Aldrich). The amount of cellular RNA was normalized to the cell number and corrected for purification efficiencies. Then quantitative northern blotting (Li-COR imaging system) was performed to determine the number of Y RNA molecules in HEK293 cells. Each Y RNA was quantified using *in vitro* transcribed RNA as reference. Y RNA quantification was repeated three times and cellular Y RNA copies were calculated resulting in 12 data points per Y RNA. The results of 12 quantifications are shown on the left; the mean number of cellular Y RNAs is depicted on the right. Representative images of the resulting northern blots are depicted in Supplementary Figure S1. The *in vitro* transcription procedure as well as the used northern blotting protocols were described previously (Kohn et al., 2015).

NcRNAs can also act as molecular decoys of RBPs and thereby modulate their RNA-binding (Figure 1). This sequestering is either achieved by a direct competition with other RNA substrates or by forcing the respective RBP in an ‘inactive’ conformation (e.g. P-TEFb in the 7SK-RNP). Irrespective of the molecular basis, this sequestering stalls RBPs in a dormant state. Importantly, the decoy role of ncRNAs implies that the potency of regulation strongly depends on the concentration of participating factors, ncRNAs and RBPs, as well as the affinity and stability of their association. In support of this, examples for decoys have only been described for few, highly abundant ncRNAs like Y RNAs. These can reach exceedingly high cellular concentrations. We quantified the approximate number of Y RNA molecules in HEK293 cells by quantitative northern blotting using *in vitro* transcribed Y RNAs as reference (Figure 2; Supplementary Figure S1). According to our calculations, ~8.14 × 10^5^ Y RNA molecules are present in a HEK293 cell with nearly 2 × 10^5^ molecules of Y3 RNA alone. This implicates that highly abundant small ncRNAs like Y3 are able to act as molecular RBP decoy.

A prominent example for POLIII-transcripts that act as decoy factors is the RNP formed by 7SK. This ncRNA is ~330 nts in length and associates with the La protein right after synthesis (Chambers et al., 1983). The methylphosphate capping enzyme MEPCE associates with and modifies the 5′-end of 7SK, which renders the RNA more stable and triggers the release of La and its replacement by the La-related protein LARP7 (Muniz et al., 2013). Subsequently, RBPs of the HEXIM-family (HEXIM1/2) can bind to 7SK. This results in the ‘activation’ of the previously dormant RNP and promotes the 7SK-RNP-dependent inhibition of transcription elongation by POLII. This inhibitory function is facilitated by the HEXIM-proteins that, solely when associated with 7SK, associate with the transcription elongation factor P-TEFb, consisting of CDK9 and Cyclin T1. Productive transcription elongation is dependent on P-TEFb activity, since its kinase inactivates negative regulators like NELF and DSIF. The latter facilitate promoter-proximal pausing of transcription (Adelman and Lis, 2012). Furthermore, P-TEFb can directly phosphorylate POLII-CTD at serine-2 to promote productive transcript elongation (Marshall et al., 1996). By sequestration of P-TEF-b, the 7SK RNP can potently inhibit transcription elongation and thereby serves as important negative regulator of RNA-synthesis in general. However, the association of hnRNPs (A1, A2/B1, Q, and R) and RNA helicase A with 7SK, as well as the phosphorylation of HEXIM-proteins, can trigger the release of P-TEF-b to allow again productive transcription (AJ et al., 2016).

The 7SK RNP highlights some of the criteria a real decoy has to meet. First, the decoy RNP has to be sufficiently abundant to act as an efficient competitor for the respective target proteins. Indeed, 7SK is an abundant nuclear RNA and it was previously shown that 50%–90% of cellular P-TEFb is constantly associated with 7SK depending on the cell type (Nguyen et al., 2001; Yang et al., 2001; Kim et al., 2011). Second, mechanisms for the control of decoy activity and/or its regulated release are required. In case of the 7SK RNP three modes of regulation have been revealed. The release of P-TEFb can be induced by post-translational modifications or by a reduction of 7SK levels due to altered RNA stability controlled via MEPCE-directed capping. Furthermore, various RBPs (e.g. hnRNPs) compete with P-TEF-b for 7SK-binding and thus influence the P-TEF-b occupancy of 7SK (AJ et al., 2016). These properties of 7SK-directed RNP function highlight that the activity and regulation of ncRNA decoys is highly versatile and can be fine-tuned by a variety of regulatory mechanisms.

**Figure 3:**
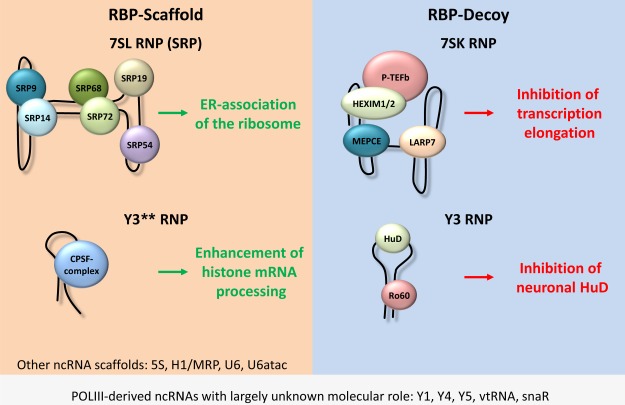
RNPs assembled on POLIII-derived ncRNA and their functions. NcRNAs act in concert with RBPs to exert a variety of cellular functions. POLIII-derived ncRNAs can either act as scaffolds (e.g. 7SL and Y3**) or as decoys (e.g. 7SK and Y3). The function of other ncRNA scaffold is not outlined here (e.g. 5S or U6). Note that although some POLIII-derived ncRNAs were identified long ago, a precise molecular role was not yet identified (e.g. vtRNAs and snaRs).

## Versatile roles of Y RNAs—cytoplasmic RBP decoys and nuclear scaffolding factors

Y RNAs are small POLIII-derived ncRNAs that are highly conserved in vertebrates. They form nuclear as well as cytoplasmic RNPs with the major protein determinants Ro60 and La, as well as additional RBPs (Kohn et al., 2013). In human four different full length Y RNAs have been described: Y1 (112 nts), Y3 (101 nts), Y4 (93 nts), and Y5 (83 nts) (Hendrick et al., 1981; Lerner et al., 1981). From these, smaller transcripts can be derived, for instance the Y3** ncRNA that terminates at U60/61 of its precursor (Wolin and Steitz, 1983). All four human Y RNAs precursors are characterized by a typical stem-loop structure and an oligo-uridine terminator (Kohn et al., 2013). Y RNAs associate with a variety and partially distinct set of nuclear, as well as cytoplasmic RBPs, suggesting overlapping and diverse functions of Y RNAs. Previously, it was determined that the Y RNPs range in size from 150 to 550 kDa (Fabini et al., 2000). Therefore, it is assumed that a single Y RNA molecule can associate with more than one protein simultaneously. Consistent with their high abundance and the plethora of potential binding partners reported to date Y RNAs are *bona fide* examples of decoying POLIII-synthesized ncRNAs that as well can act as scaffolds in the assembly of regulatory RNPs, as previously proposed (Kohn et al., 2013).

Most recently, it was reported that Y3 associates with the human antigen D (HuD), also named embryonic lethal abnormal vision like 4 (ELAVL4) (Tebaldi et al., 2018). HuD plays a crucial role in controlling neuronal cell fate by regulating the alternative splicing, alternative polyadenylation, localization, turnover, and translation of neuronal mRNAs (Bronicki and Jasmin, 2013). The overexpression of HuD in mouse motor neuron-like cells (NSC-34) increases the global translation efficiency in an mTORC1-dependent manner. Crosslinking and cDNA analysis (CRAC) as well as RNA pulldown experiments confirmed that Y3 RNA associates with HuD in NSC-34 cells. The Y3 RNA comprises two HuD binding sites in its loop-region (Tebaldi et al., 2018). Interestingly the analysis of the average molecule numbers of HuD (2.13 × 10^5^ molecules) and Y3 RNA (1.09 × 10^5^ molecules) uncover a 1.95 ratio between the protein and the RNA in NSC-34 cells, given that two different HuD molecules associate with both binding regions of Y3. Therefore, Y3 RNA can potentially sequester all HuD molecules from potential mRNA targets when assuming exclusive association with HuD. In fact it was shown that Y3 competes for HuD association with the target mRNAs Eif4a1, Eef1a1 and Ncam1. Accordingly the overexpression of Y3 RNA decreases the protein levels of Eif4a1 and Eef1a1 (Tebaldi et al., 2018). Sucrose gradient analysis showed a shift of HuD from sub-polysomal fractions to the heavy polysomes upon Y3 depletion, indicating enhanced translation of HuD targets. Consistent with these results an increased polysomal localization of the HuD target mRNA EiF4a2 was detected upon Y3 depletion. This mechanism represents a remarkable example for the function of POLIII-derived ncRNAs acting as RBP-decoy (Figure 3). According to this model, the Y3-directed sequestering of HuD antagonizes its mRNA-binding, impairs the translation of specific mRNAs and consequently interferes with neuronal differentiation.

In contrast to the mainly cytoplasmic role of the Y3 precursor, its processed Y3** was reported to serve scaffolding functions promoting the 3′-end processing of replication-dependent histone mRNAs in the nucleus (Kohn et al., 2015). The Y3** ncRNA associates with pre-mRNA processing factors of the CPSF (cleavage and polyadenylation specificity factor) family and assembles a minimal processing complex in a FIP1L1-dependent manner. Consistent with a pivotal role of uridine-rich 3′-ends of POLIII-synthesized ncRNAs in directing nuclear retention and presumably function the association of Y3** with 3′-end processing factors strictly relies on a uridine-rich stretch at its 3′-end. The depletion of Y3/Y3** selectively impairs the 3′-end processing of histone pre-mRNAs and reduces the apparent size of histone locus bodies (HLBs). These indicate discrete nuclear foci of histone mRNA synthesis and processing (Duronio and Marzluff, 2017). Although the exact mechanism of how Y3**–CPSF complexes are recruited to HLBs remains unknown, fluorescent recovery after photobleaching (FRAP) revealed that Y3**, but not Y3, is essential for the recruitment of the CPSF to HLBs (Kohn et al., 2015). These findings suggest that the Y3** ncRNA is a scaffolding factor promoting the assembly of minimal processing complexes and facilitates their recruitment to HLBs where they direct the correct processing of histone mRNAs (Figure 3).

## Summary and conclusion

Recent findings support the role of previously described POLIII-derived ncRNAs as central scaffolding and decoy factors guiding the function of RNPs in the control of gene expression at various levels. In support of their only partially revealed fundamental roles, the expression, structure, as well as major associating RBPs of most to date studied POLIII-derived ncRNAs are highly conserved. The most prominent example of scaffolding POLIII-generated ncRNAs is the 7SL RNA promoting ER-associated mRNA translation in the cytoplasm. In the nucleus, the Y3-derived Y3** RNA was shown to promote the assembly and HLB-recruitment of 3′-end processing factors to guide the correct 3′-end processing of histone mRNAs. Additional well-known ncRNAs like 5S (ribosome assembly), H1/MRP (tRNA processing), and U6/U6atac (spliceosome assembly) serve essential roles in scaffolding the assembly of functional RNPs. In addition to the mainly reported scaffolding function, recent findings uncovered novel functions of POLIII-derived transcripts in their role as protein decoys. The best studied example, the 7SK ncRNA, serves as an inhibitor of POLII-mediated transcription by sequestering P-TEFb that promotes productive transcript elongation by POLII. Although previously proposed, only recently, Y3 was identified to interfere with HuD-dependent control of mRNA translation by sequestering this RBP in Y RNPs. Notably, the diverse roles of Y3 and Y3** suggest that even rather small ncRNAs can give rise to functional diverse RNA isoforms serving roles in the nucleus and/or cytoplasm. Future studies have to explore the roles of the plethora of POLIII-derived ncRNAs suggested by RNA-sequencing approaches. These studies will have to identify associated proteins, subcellular localization, and most importantly cellular abundance. This will set the stage to further explore if ncRNAs of unknown cellular function, e.g. vtRNAs or snaRs, act as scaffolds, decoys, or both (Figure 3). Most likely, some of these ncRNAs act in a tissue and/or cell type-specific manner. For instance, snaRs were shown to be mainly expressed in testis and brain, which might point toward a tissue restricted function (Parrott et al., 2011). Y3 on the other hand is expressed ubiquitously but the expression of its regulated partner HuD is restricted to neuronal tissue. Therefore, especially the identification of ncRNA decoys remains challenging, since the physiological relevance of decoy functions essentially relies on the concentration of the decoying ncRNAs as well as its major target(s). Although barely explored and understood, previous studies suggested that some POLIII-derived ncRNAs are implicated in human diseases. 7SK RNA for instance was shown to be decreased in human cancer cells (Abasi et al., 2016). The tumorsupressive role of this ncRNA likely depends on its function to restrict POLII transcription. Y RNAs associate with the Ro60 protein, a major autoantigen triggering autoimmunity in lupus (Greiling et al., 2018). Moreover, Y RNA expression appears deregulated in cancer, suggesting that these ncRNAs modulate the activity of oncogenic or tumorsupressive RBPs (Christov et al., 2008).

## Funding

This work was supported by the German Research Foundation (DFG; research projects GRK1591 and SPP1935).

**Conflict of interest**: none declared

## Supplementary Material

Supplementary_Figure_mjz049
