# Supplementary material for: POLIII-derived non-coding RNAs acting as scaffolds and decoys
Source: J Mol Cell Biol. 2019 Jun 1;11(10):880–5. doi: 10.1093/jmcb/mjz049 (PMC6884708; doi:10.1093/jmcb/mjz049)
Supplement: Supplementary_Figure_mjz049 [file supplementary_figure_mjz049.pdf]

Supplementary Figure

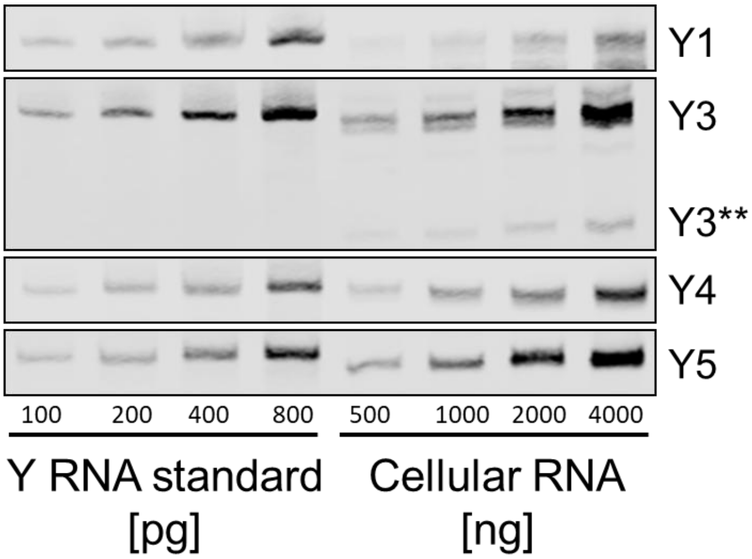

The Y RNA level in HEK293 cells were assessed by quantitative Northern Blot. *In vitro* transcribed Y RNAs were used as reference and cellular Y RNAs were quantified by fluorescence imaging. Representative Northern Blot images are shown for each Y RNA. Quantifications were repeated three times resulting in twelve data points for cellular Y RNA level.
